# Oncotherapy resistance explained by Darwinian and Lamarckian models

**DOI:** 10.1172/JCI179788

**Published:** 2024-04-15

**Authors:** Yogen Saunthararajah

**Affiliations:** Department of Translational Hematology and Oncology Research, Taussig Cancer Institute, Cleveland Clinic, Cleveland, Ohio, USA.

## Abstract

Cell and antibody therapies directed against surface molecules on B cells, e.g., CD19-targeting chimeric antigen receptor T cells (CD19 CAR-T), are now standard for patients with chemorefractory B cell acute lymphoblastic leukemias and other B cell malignancies. However, early relapse rates remain high. In this issue of the *JCI*, Aminov, Giricz, and colleagues revealed that leukemia cells resisting CD19-targeted therapy had reduced CD19 but also low CD22 expression and were sensitive to Bruton’s tyrosine kinase and/or MEK inhibition. Overall, their observations support the evolution of resistance following a Lamarckian model: the oncotherapy directly elicits adaptive, resistance-conferring reconfigurations, which are then inherited by daughter cells as epigenetic changes. The findings prompt reflection also on the broader role of epigenetics in decoupling of replication from lineage differentiation activation by the B cell lineage master transcription factor hub. Such oncogenesis and resistance mechanisms, being predictable and epigenetic, offer practical opportunities for intervention, potentially non-cross-resistant and safe vis-à-vis present cytotoxic and CAR-T treatments.

## How malignant B cells resist CAR-T therapies

Cell and antibody therapies directed against surface molecules on B cells, e.g., CD19-targeting chimeric antigen receptor T cell therapy (CD19 CAR-T), are now standard treatments for patients with chemorefractory B cell acute lymphoblastic leukemias (B-ALL) and other B cell malignancies. However, upfront treatment failure and early relapse rates range from 20% to 60% (1, 2). In this issue of the *JCI*, Aminov, Giricz, and co-authors found that B-ALL cell lines expanding through CD19-targeted therapy expressed lower levels of CD19 RNA and protein than did parental cells, observations they extended to nine pediatric patients with B-ALL relapsing after CD19 CAR-T (3). This finding in and of itself was unsurprising, since CD19 loss had already been described in clinical trials of CD19 CAR-T to treat B-ALL (4, 5). There was, however, another reduction. Compared with parental cells, B-ALL cells resistant to CD19-targeted therapy, in the patients as well as in vitro, expressed less CD22, even though there was no CD22-targeted treatment to select for such suppression. This observation has immediate clinical relevance, since CD22 CAR-T, as well as dual-targeting CD19/CD22 CAR-T, is in clinical trials as an alternative or complement to CD19 CAR-T (6) — the results from Aminov, Giricz, and co-authors predict CD22 CAR-T are unlikely to salvage CD19 CAR-T resistance (3).

## Candidate targets for salvage therapy

Fortunately, Aminov et al. revealed alternative candidate targets. The B cell receptor (BCR) signals via Bruton’s tyrosine kinase (BTK) and dictates maturation, proliferation, and life and death of cells committed to the B cell lineage (7, 8). Aminov, Giricz, and colleagues found that malignant B cells resisting CD19-targeted therapy preserved or upregulated BCR components and BTK, even as they downregulated CD19 and CD22 expression (3). These observations have functional and potentially therapeutic implications. B-ALL cells resistant to CD19-targeted treatment were several-fold more sensitive to the growth inhibitory effects of small-molecule inhibitors against BTK and downstream MEK than were parental B-ALL cells. Importantly, several BTK and MEK inhibitors have been approved by the FDA, although not to treat B-ALL.

## Darwin and Lamarck

**Figure 1:**
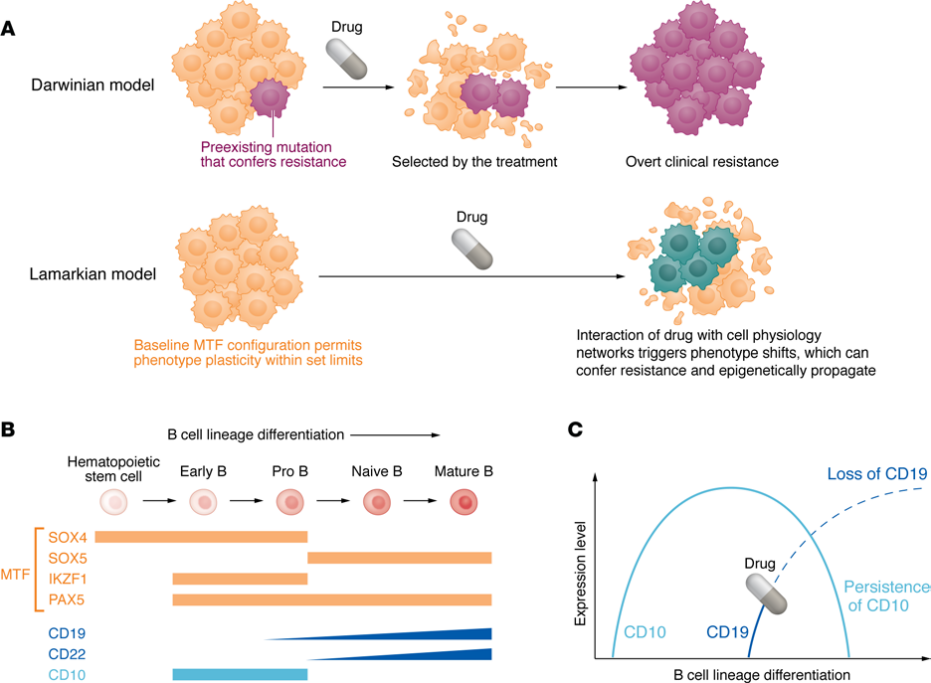
Phenotype of B cell malignancies at diagnosis and resistance depends on the expression of B cell lineage MTFs during different stages of B cell lineage differentiation. (**A**) In B cell malignancies, treatment resistance occurs via two evolutionary models: A Darwinian model for treatment resistance occurs when a preexisting mutation confers resistance and is selected for during treatment, e.g., selection by chemotherapy for *TP53* mutations and/or deletions. Alternatively, a Lamarckian model predicts that an oncotherapeutic can directly trigger adaptive responses in malignant cells. The baseline MTF configuration of the cells constrains the range of adaptive shifts, which are propagated to daughter cells via epigenetic mechanisms. Notably, both models can act concurrently. (**B**) Coordinated shifts in the expression of lineage MTFs and surface receptors occur during normal B cell lineage differentiation. Treatments that target CD19 or BTK affect inputs into the lineage MTF circuit and may alter malignant phenotypes in a pattern dictated by the baseline MTF configuration and B cell lineage differentiation. (**C**) CD10 and CD19 are expressed at different stages in B cell lineage differentiation and present targeting opportunities. B cells undergoing differentiation that have been targeted by CD19 treatments may shift phenotypes to confer resistance but remain susceptible to alternative targeted therapies such as anti-CD10.

Selection by chemotherapy or CAR-T for malignant B cells containing inactivating mutations and/or deletions of key apoptosis/cell death genes, e.g., *TP53* (encoding p53), *CDKN2A* (encoding p16), and *PMAIP1* (encoding NOXA), exemplifies Darwinian processes in resistance onset and propagation (1) (Figure 1). However, coordinated downregulation of CD19 and CD22, simultaneous with BCR and BTK preservation or upregulation, seems an unlikely consequence of random or accidental genetic events. That is, an exclusively Darwinian model, especially over the short time-scales observed experimentally by Aminov et al., seems unlikely, and another process must explain how these phenotype changes emerge and stabilize. CD19 is normally activated upon hematopoietic stem cell commitment into the B cell lineage, and then progressively increases in expression with onward B cell lineage maturation. It functions as a coreceptor or accessory to the BCR/BTK pathway, signaling into B cell lineage cells via the PI3K/AKT pathway, and in this way contributes to B-lineage differentiation and specialization (7–9). Given this normal function of CD19, insofar as malignant B cells survive weapon payloads attached to CD19-targeting treatments, the CD19 targeting itself will affect the cellular phenotype, either by the direct inhibition of CD19 function, or indirectly through the selection of cells on the lower end of the CD19 expression spectrum (Figure 1). The state of having less CD19 function and expression can be expected to favor maturation arrest early in the B cell lineage differentiation continuum, when CD19 and CD22 expression is lower but BCR and BTK expression is preserved (Figure 1). That this is the case is also supported by the observation by Aminov et al. that the master transcription factor (MTF) SOX4 was upregulated in the resistant cells (Figure 1). Viewed in this light, other predictions can be made, e.g., CD10 could be a potential alternative to CD22 as a surface target for salvage therapy — CD10-targeting CAR-T is being explored (10) (Figure 1).

Lamarck proposed directed evolution such that the environment instructs pro-fitness modifications (aka adaptation) heritable by subsequent generations (11) (Figure 1). Although it is difficult to transmit adaptation occurring at a somatic level into a separate germline compartment, unicellular neoplastic evolution faces no such difficulties because adaptive shifts in gene expression are readily propagated to daughter cells via epigenetic inheritance. Moreover, persistence of the environmental cue, e.g., CD19 CAR-T, can be expected to reinforce the adaptations in daughter cells, since these shifts after all emerge organically and predictably from cell physiology networks. Aminov, Giricz, and colleagues found that the suppression of CD19 and CD22 was indeed an epigenetic, Lamarckian process and not genetic (3). There is, thus, a further therapeutic implication: epigenetic enzymes mediating repression of CD19 and CD22 also constitute candidate targets for salvage therapy.

## The larger context

It would be tunnel-visioned, however, to ignore that, in fact, hundreds of B cell lineage differentiation genes are aberrantly epigenetically repressed to result in B-ALL in the first-place. Even apparently so-called mature B cell malignancies, such as chronic lymphocytic leukemia and multiple myeloma, display aberrant epigenetic repression of final B cell specialization programs (12–15). A few of the TFs expressed in cells are MTFs that collaborate in hubs to govern the expression of other TFs and thousands of genes, thereby dictating cell fates and functions (16). A central, deterministic role for the B cell lineage MTF hub explains why, confusingly, it serves dual functions as a tumor suppressor and as an oncogene in B cell lineage transformation. The tumor suppressor role is shown by partial loss of the hub’s function, e.g., by inactivating mutations and/or deletions of individual lineage MTFs, e.g., *IKZF1* or *PAX5*. Additionally, epigenetic enzymes that lineage MTFs recruit in order to remodel lineage differentiation genes for activation — coactivators — are recurrently mutated and deleted (17, 18). Conversely, genes encoding for repressing enzymes in the lineage MTF hub, also known as corepressors, may be amplified. These genetic alterations impair the ability of the lineage MTF hub to couple high-grade replications with lineage-committed differentiation. Stated another way, imbalances among the hub’s corepressors and/or coactivators preserve the activation of replication genes, which are constitutively accessible — in other words, open on Sundays! — but repress lineage differentiation genes, which do require chromatin remodeling for activation (19–22). On the other hand, these hubs are similar to oncogenes, in that the residual lineage MTFs in the hub are cancer addictions upon which malignant B cells depend to exist and replicate (23, 24).

## Conclusions

In clinical reality, salvage treatments for refractory and relapsed malignancy should offer not just a non-cross-resistant pathway of action vis-à-vis preceding failed treatments, but also better safety, since patients’ physiologic reserves may well be depleted by everything they have already gone through. The observations by Aminov, Giricz, and colleagues related to resistance to CD19-targeted treatment provide practical, useful guidance, in that they point to alternative molecularly targeted, nonchemotherapeutic options for salvage therapy that can immediately be repositioned (3). Epigenetic inheritance of these resistance mechanisms prompts a widening of perspective to recognize the central role of aberrant epigenetic repression of lineage differentiation genes in causing malignant self-replication — the beating heart of malignancy — resistant or otherwise. This is a relatively neglected but druggable space that offers non-cross-resistance with chemotherapy and CAR-T therapies. Chemotherapy and CAR-T treatments must harness key apoptosis and cell death genes, such as *TP53*, *CDKN2A*, and *PMAIP1*, in order to terminate malignant replications. Thus, in a Darwinian process, malignant B cells that are primary-refractory or relapsed after such treatments may contain inactivating mutations or deletions of these genes (1). Restoring the ability of malignant B cells to activate onward lineage differentiation programs forces cell-cycle exits even if key apoptosis/cell death genes are deleted and absent, meaning that treatments that inhibit repressing epigenetic enzymes to thereby resume lineage maturations can be non-cross-resistant with chemotherapy or CAR-T (19, 22). Moreover, some drugs that do this, e.g., decitabine to deplete DNMT1, can be used in ways that are not cytotoxic to normal dividing cells, including normal immune cells that are utilized by immunotherapies and autologous CAR-T therapies (19, 25). There are, thus, untapped opportunities to remedy mechanisms of resistance and even root-cause malignant self-replication that are distinct from treatment conventions attempting to impose cell death.

## Version 1 04/15/2024

Electronic publication
